# Supplementary material for: The racialization of pit bulls: What dogs can teach us about racial politics
Source: PLoS One. 2024 Jun 28;19(6):e0305959. doi: 10.1371/journal.pone.0305959 (PMC11213322; doi:10.1371/journal.pone.0305959)
Supplement: S1 Appendix — (DOCX) [file pone.0305959.s001.docx]

**Appendix**

***Coding of Explanatory Variables***

Blacks Favorability: A five-category variable recoded from 0 (very unfavorable) to 1 (very favorable). Don’t knows are coded as .5.

Dog Favorability: A five-category variable recoded from 0 (very unfavorable) to 1 (very favorable). Don’t knows are coded as .5.

Education: A five-category variable recoded from 0 (No high school diploma) to 1 (post-grad degree)

Male: An indicator variable taking on a value of 1 (male) or 0 (all other responses)

Old Fashioned Racism: A nine-category, two-item additive index recoded from 0 (lowest) to 1 (highest). The two items asked respondents how strongly they agreed or disagreed (5-cateogry) with the following statements (1) “I prefer that my close relatives marry spouses of their same race,” (2) “I think it's alright for Blacks and Whites to date each other” (reverse coded)

Partisanship: A seven-category variables recoded from 0 (strong Democrat to 1 (strong Republican)

Racist Google Searches (State-level). Measured by Google Trends with the prevalence of state-level searches for “nigger” from 2004-2019. Recoded from 0 (lowest state score) to 1 (highest state score).

Violent: Black Men: A five-category variable recode from 0 (violent describes group “not at all well”) to 1 (violent describes group “extremely well”).

Violent: Black Women: A five-category variable recode from 0 (violent describes group “not at all well”) to 1 (violent describes group “extremely well”).

Violent: Muslim Men: A five-category variable recode from 0 (violent describes group “not at all well”) to 1 (violent describes group “extremely well”).

Violent: Muslim Women: A five-category variable recode from 0 (violent describes group “not at all well”) to 1 (violent describes group “extremely well”).

Violent: White Men: A five-category variable recode from 0 (violent describes group “not at all well”) to 1 (violent describes group “extremely well”).

Violent: White Women: A five-category variable recode from 0 (violent describes group “not at all well”) to 1 (violent describes group “extremely well”).

White Opposition to Interracial Dating (State-level): Measured in the 1987-2012 Pew Values Survey Merged File with the average level of white residents’ disagreement with the statement: “I think it's alright for Blacks and Whites to date each other.” Recoded from 0 (lowest state score) to 1 (highest state score)

White Republican Partisanship (State-level): Measured in the 1987-2012 Pew Values Survey Merged File with the average white residents’ 5-category partisanship, ranging from 1 (Democrat) to 5(Republican). Recoded from 0 (lowest state score) to 1 (highest state score)

Whites Favorability: A five-category variable recoded from 0 (very unfavorable) to 1 (very favorable). Don’t knows are coded as .5.

***Coding of Dependent Variables***

Pit Bull Adoption: An indicator variable taking on a value of 1 (would consider adopting a pit bull) or 0 (all other responses).

Pit Bull Aggressiveness: An indicator variable taking on a value of 1 (pit bulls not naturally more aggressive than other breeds) or 0 (all other responses).

Pit Bull Legality: An indicator variable taking on a value of 1 (should be legal to own a pit bull) or 0 (all other responses)

Pit Bull Safety: An indicator variable taking on a value of 1 (it’s safe for pit bull to live in residential areas) or 0 (all other responses).

Net Favorability Dog Scale: A nine-item additive index of net favorability ratings (-1 to 1) of the following: Dogs, Labradors, Golden Retrievers, German Shepherds, Border Collies, Huskies, Bulldogs, Dalmatians and Chihuahuas. The scale is recoded from 0 (rate all of the breeds unfavorably) to 1 (rate all of the breeds favorably).

Net Favorability of Pit Bulls: A three-category variable recoded from -1 (rate pit bulls very/ somewhat unfavorably to 1 (rate pit bulls very/somewhat favorably).

**Information on Lucid Recruitment and Informed Consent**

At the start of each one of the Lucid surveys commissioned participants first received the following information, which explained the purposes of the study, that participation is both voluntary and confidential, that respondents will be compensated for their time and that there is not penalty for withdrawing.

*Study Information Provided at the Start of Every Survey*

You are being asked to take part in a research study. The study seeks to understand your views on several social and political issues. Please answer each survey question to the best of your ability. To take part in the study you must be 18 or over and a U.S. resident.

The survey will take no more than 15 minutes of your time.


There are no foreseeable risks to you if you take part in this study. 


You have been selected to be in this study through Lucid. If you complete the survey, you will be provided with a payment to compensate you for your time. Participation in this study is voluntary. There is no penalty for withdrawing.


Your participation in this study is confidential. Gathered data will not have any identifiable information. 


Study contacts: If you have any questions about this research project, please contact the principal investigator, Nathan Kar Ming Chan (nkchan@uci.edu). If you have questions about your

rights or welfare as a participant, please contact the University of California Irvine Institutional

Review Board (IRB) Office, at (949) 824-6662 or by email at IRB@research.uci.edu.

Table A1: (Logistic Regression) Predictors of White Americans’ Opinions about Pit Bulls

| Not More Aggressive Would Adopt Safe for Residential Legal to Own  CCES Lucid CCES Lucid CCES Lucid CCES Lucid | | | | | | | | | | | | | | | | | | | | | | | | | | | | | | | | | | | | | | | | | | | | | |  |  |  |
| --- | --- | --- | --- | --- | --- | --- | --- | --- | --- | --- | --- | --- | --- | --- | --- | --- | --- | --- | --- | --- | --- | --- | --- | --- | --- | --- | --- | --- | --- | --- | --- | --- | --- | --- | --- | --- | --- | --- | --- | --- | --- | --- | --- | --- | --- | --- | --- | --- |
|  | | |  | | | |  | | | |  | |  | | | | | | | |  | | | |  | | | | |  |  |  |  |  |  |  |  |  |  |  |  |  |  |  |  |  |  |  |
| Black Favorability | | | | 1.20^***^  (.317) | | | | | .787^***^  (.108) | | | | | |  | | | .801^*^  (.323) | | | | | .630^***^  (.110) | | | |  | | | | 1.28^***^  (.321) | | | 1.07^***^  (.198) | | |  | | | 1.36^***^  (.365) | | | .849^***^  (.110) | | | | |  |
|  | | | |  | | | | |  | | | | | |  | | |  | | | | |  | | | |  | | | |  | | |  | | |  | | |  | | |  | | | | |  |
| Constant | | | | -.415  (.259) | | | | | -.247^**^  (.084) | | | | | |  | | | -.936^**^  (.270) | | | | | -.711^***^  (.086) | | | |  | | | | -.910^**^  (.267) | | | -.703^***^  (.154) | | |  | | | -.273  (.263) | | | .020  (.084) | | | | |  |
|  | | | |  | | | | |  | | | | | |  | | |  | | | | |  | | | |  | | | |  | | |  | | |  | | |  | | |  | | | | |  |
|  | | | |  | | | | |  | | | | | |  | | |  | | | | |  | | | |  | | | |  | | |  | | |  | | |  | | |  | | | | |  |
| Observations | | | | 742 | | | | | 4560 | | | | | |  | | | 743 | | | | | 4559 | | | |  | | | | 741 | | | 1437 | | |  | | | 740 | | | 4560 | | | | |  |
| Pseudo R^2^ | | | | | | .016 | | | .009 | | | | | |  | | | .007 | | | | | .006 | | | |  | | | .017 | | | | .015 | | |  | | | .020 | | | .010 | | | | |  |
|  | | | |  | | | | |  | | | | | |  | | |  | | | | |  | | | |  | | | |  | | |  | | |  | | |  | | |  | | | | |  |
| ------------------------ | | | |  | | | | |  | | | | | |  | | |  | | | | |  | | | |  | | | |  | | |  | | |  | | |  | | |  | | | | |  |
| Old Fashioned Racism | | | | -1.15^***^  (.288) | | | | | -1.36^***^  (.114) | | | | | | |  | | | -.1.27^*^  (.298) | | | | | -.899^***^  (.113) | | | |  | | | | -1.83^***^  (.300) | | | -1.37^***^  (.201) | | |  | | | -1.91^***^  (.305) | | | -1.34^***^  (.117) | | | | |
|  | | | |  | | | | |  | | | | | |  | | |  | | | | |  | | | |  | | | |  | | |  | | |  | | |  | | |  |  |  |  |  |  |
| Constant | | | | .868^***^  (.118) | | | | | .804^***^  (.059) | | | | | |  | | | .062  (.111) | | | | | .059  (.048) | | | |  | | | | .623^***^  (.115) | | | .540  (.087) | | |  | | | 1.39^***^  (.131) | | | 1.11^***^  (.054) | | | | |  |
|  | | | |  | | | | |  | | | | | |  | | |  | | | | |  | | | |  | | | |  | | |  | | |  | | |  | | |  | | | | |  |
| Observations | | | | 739 | | | | | 4560 | | | | | |  | | | 740 | | | | | 4559 | | | |  | | | | 738 | | | 1437 | | |  | | | 737 | | | 4560 | | | | |  |
| Pseudo R^2^ | | | | | | | .018 | | | .024 | | | | | |  | | | .020 | | | | | .011 | | | |  | | | .042 | | | | .025 | | |  | | | .048 | | | .023 | | | | |
|  | | | |  | | | | |  | | | | | |  | | |  | | | | |  | | | |  | | | |  | | |  | | |  | | |  | | |  | | | | |  |
|  |  |  | | | |  | | | |  | |  | | | | | | | |  | | | | | | | | | | | | | | | | | | | | | | | | |  |  |  |  |

Significance codes: ^+^p < .10, ^*^p < .05, ^**^p < .01, ^***^p < .001

Note: Dependent variables are recoded from 0-1, with 1 being the pro-pit position and 0 representing all other responses. CCES utilize post-stratification weights, but Lucid does not provide weights with its raw data. Sources: Pooled Lucid Surveys 2019-2021; Lucid Survey; 2018 CCES Team Module; white respondents only.

Table A2: (Logistic Regression) Predictors of White Americans’ Opinions about Pit Bulls

| Not More Aggressive Would Adopt Safe for Residential Legal to Own  CCES Lucid CCES Lucid CCES Lucid CCES Lucid | | | | | | | | | | | | | | | | | | | | | | | | | | | | | | | | | | | | | | | | | |  |  |  |
| --- | --- | --- | --- | --- | --- | --- | --- | --- | --- | --- | --- | --- | --- | --- | --- | --- | --- | --- | --- | --- | --- | --- | --- | --- | --- | --- | --- | --- | --- | --- | --- | --- | --- | --- | --- | --- | --- | --- | --- | --- | --- | --- | --- | --- |
|  | | |  | | |  | | | |  | |  | | | | | |  | | |  | | | |  |  |  |  |  |  |  |  |  |  |  |  |  |  |  |  |  |  |  |  |
| Black Favorability | | | | 1.46^***^  (.364) | | | .907^***^  (.118) | | | | | |  | | .995^**^  (.376) | | | | .751^***^  (.123) | | | |  | | | 1.45^***^  (.375) | | | 1.08^***^  (.220) | | |  | | | 1.60^***^  (.367) | | | .926^***^  (.121) | | | | |  |  |
|  | | | |  | | |  | | | | | |  | |  | | | |  | | | |  | | |  | | |  | | |  | | |  | | |  | | | | |  |  |
| White Favorability | | | | -.965^*^  (.449) | | | -.376^**^  (.140) | | | | | |  | | -.908^*^  (.418) | | | | .108  (.139) | | | |  | | | -.684  (.43)1 | | | .229  (.249) | | |  | | | -.600  (.462) | | | .137  (.144) | | | | |  |  |
|  | | | |  | | |  | | | | | |  | |  | | | |  | | | |  | | |  | | |  | | |  | | |  | | |  | | | | |  |  |
| Party Identification | | | | -.316  (.245) | | | -.271^**^  (.082) | | | | | |  | | -.162  (.245) | | | | .043  (.085) | | | |  | | | -.519^*^  (.243) | | | .151  (.155) | | |  | | | -.278  (.253) | | | -.025  (.086) | | | | |  |  |
|  | | | |  | | |  | | | | | |  | |  | | | |  | | | |  | | |  | | |  | | |  | | |  | | |  | | | | |  |  |
| Actual Age | | | | -.034^***^  (.005) | | | -.022^***^  (.002) | | | | | |  | | -.045^***^  (.005) | | | | -.046^***^  (.002) | | | |  | | | -.045^***^  (.005) | | | -.045^***^  (.004) | | |  | | | -.035^***^  (.005) | | | -.031^***^  (.002) | | | | |  |  |
|  | | | |  | | |  | | | | | |  | |  | | | |  | | | |  | | |  | | |  | | |  | | |  | | |  | | | | |  |  |
| Education | | | | -1.24^***^  (.312) | | | -.270^*^  (.123) | | | | | |  | | -.730^*^  (.311) | | | | -.133  (.127) | | | |  | | | -.602^+^  (.307) | | | .819^**^  (.253) | | |  | | | -.544^+^  (.317) | | | .104  (.130) | | | | |  |  |
|  | | | |  | | |  | | | | | |  | |  | | | |  | | | |  | | |  | | |  | | |  | | |  | | |  | | | | |  |  |
| Male | | | | -.227  (.170) | | | -.038  (.062) | | | | | |  | | -.352^*^  (.170) | | | | -.063  (.065) | | | |  | | | -.243  (.169) | | | .010  (.114) | | |  | | | .020  (.176) | | | .101  (.066) | | | | |  |  |
|  | | | |  | | |  | | | | | |  | |  | | | |  | | | |  | | |  | | |  | | |  | | |  | | |  | | | | |  |  |
| Constant | | | | 2.83^***^  (.538) | | | 1.39^***^  (.161) | | | | | |  | | 2.48^***^  (.525) | | | | 1.39^***^  (.160) | | | |  | | | 2.48^***^  (.528) | | | .833^**^  (.290) | | |  | | | 2.27^***^  (.544) | | | 1.33^***^  (.168) | | | | |  |  |
|  | | | |  | | |  | | | | | |  | |  | | | |  | | | |  | | |  | | |  | | |  | | |  | | |  | | | | |  |  |
| Observations | | | | 740 | | | 4547 | | | | | |  | | 741 | | | | 4546 | | | |  | | | 739 | | | 1435 | | |  | | | 738 | | | 4547 | | | | |  |  |
| Pseudo R^2^ | | | | | .099 | | | | .040 | | | | | |  | | .124 | | | | .097 | | | |  | | | .134 | | | .101 | | |  | | | .090 | | | .055 | | | | |
|  | | | |  | | |  | | | | | |  | |  | | | |  | | |  | | |  | | |  | | |  | | |  | | |  | | |  |  |  |  |  |
|  |  |  | | |  | | | |  | |  | | | | | |  | | | | | | | | | | | | | | | | | | | | | | | |  |  |  |  |

Significance codes: ^+^p < .10, ^*^p < .05, ^**^p < .01, ^***^p < .001

Note: Dependent variables are recoded from 0-1, with 1 being the pro-pit position and 0 representing all other responses. CCES utilize post-stratification weights, but Lucid does not provide weights with its raw data. Sources: Pooled Lucid Surveys 2019-2021; Lucid Survey; 2018 CCES Team Module; white respondents only.

Table A3: (Logistic Regression) Predictors of White Americans’ Opinions about Pit Bulls

| Not More Aggressive Would Adopt Safe for Residential Legal to Own  CCES Lucid CCES Lucid CCES Lucid CCES Lucid | | | | | | | | | | | | | | | | | | | | | | | | | |  |
| --- | --- | --- | --- | --- | --- | --- | --- | --- | --- | --- | --- | --- | --- | --- | --- | --- | --- | --- | --- | --- | --- | --- | --- | --- | --- | --- |
|  | | |  | | |  | | |  | |  | | | |  | |  | |  |  |  |  |  |  |  |  |
| Old Fashioned Racism | | | | -.775^*^  (.341) | | | - 1.20^***^  (.120) | | | | |  | -.594^+^  (.350) | | | -.702^***^  (.124) | |  | | -1.18^**^  (.347) | -1.28^***^  (.221) |  | -1.69^***^  (.352) | - 1.24^***^  (.124) | | |
|  | | | |  | | |  | | | | |  |  | | |  | |  | |  |  |  |  |  | | |
| Party Identification | | | | -.360  (.247) | | | -.200^*^  (.082) | | | | |  | -.226  (.248) | | | .088  (.085) | |  | | -.456^+^  (.246) | .318  (.156) |  | -.092  (.257) | .095  (.086) | | |
|  | | | |  | | |  | | | | |  |  | | |  | |  | |  |  |  |  |  | | |
| Actual Age | | | | -.031^***^  (.005) | | | -.021^***^  (.002) | | | | |  | -.043^***^  (.005) | | | -.044^***^  (.002) | |  | | -.041^***^  (.005) | -.043^***^  (.004) |  | -.029^***^  (.005) | -.028^***^  (.002) | | |
|  | | | |  | | |  | | | | |  |  | | |  | |  | |  |  |  |  |  | | |
| Education | | | | -1.24^***^  (.308) | | | -.286^**^  (.124) | | | | |  | -.719^*^  (.005) | | | -.104  (.127) | |  | | -.730^*^  (.307) | .842^**^  (.252) |  | -.817^*^  (.320) | .093  (.130) | | |
|  | | | |  | | |  | | | | |  |  | | |  | |  | |  |  |  |  |  | | |
| Male | | | | -.221  (.168) | | | -.024  (.063) | | | | |  | -.337  (.168) | | | -.050  (.065) | |  | | -.240  (.168) | -.003  (.114) |  | .040  (.176) | .123^+^  (.066) | | |
|  | | | |  | | |  | | | | |  |  | | |  | |  | |  |  |  |  |  | | |
| Constant | | | | 3.27^***^  (.362) | | | 2.05^***^  (.130) | | | | |  | 2.60^***^  (.342) | | | 2.13^***^  (.131) | |  | | 3.21^***^  (.359) | 2.00^***^  (.233) |  | 3.26^***^  (.377) | 2.33^***^  (.137) | | |
|  | | | |  | | |  | | | | |  |  | | |  | |  | |  |  |  |  |  | | |
| Observations | | | | 738 | | | 4547 | | | | |  | 739 | | | 4546 | |  | | 737 | 1435 |  | 736 | 4547 | | |
| Pseudo R^2^ | | | | .086 | | | .047 | | | | |  | .115 | | | .095 | |  | | .129 | .103 |  | .095 | .060 | | |
|  |  |  | | |  | | |  | |  | | | |  | | | | | | | | | | |  |  |

Significance codes: ^+^p < .10, ^*^p < .05, ^**^p < .01, ^***^p < .001

Note: Dependent variables are recoded from 0-1, with 1 being the pro-pit position and 0 representing all other responses. CCES utilize post-stratification weights, but Lucid does not provide weights with its raw data. Sources: Pooled Lucid Surveys 2019-2021; Lucid Survey; 2018 CCES Team Module; white respondents only.

Table A4: (Logistic Regression) Predictors of Opinions about Pit Bulls

| Not More Aggressive Would Adopt Safe for Residential Legal to Own  CCES Lucid CCES Lucid CCES Lucid CCES Lucid | | | | | | | | | | | | | | | | | | | | | | | | | | | | | | | |  |
| --- | --- | --- | --- | --- | --- | --- | --- | --- | --- | --- | --- | --- | --- | --- | --- | --- | --- | --- | --- | --- | --- | --- | --- | --- | --- | --- | --- | --- | --- | --- | --- | --- |
|  | | |  | | |  | | |  | |  | | | |  | |  | | |  |  |  |  |  |  |  |  |  |  |  |  |  |
| Black Favorability | | | | 1.17^***^  (.301) | | | .812^***^  (.099) | | | | |  | .744^*^  (.316) | | | .752^***^  (.103) | | |  | | 1.32^***^  (.320) | | 1.00^***^  (.182) | |  | | 1.53^***^  (.307) | | 1.06^***^  (.102) | | | |
|  | | | |  | | |  | | | | |  |  | | |  | | |  | |  | |  | |  | |  | |  | | | |
| White Favorability | | | | -.535^+^  (.301) | | | -.229^*^  (.105) | | | | |  | -.235  (.296) | | | .107  (.106) | | |  | | -.479  (.306) | | .124  (.186) | |  | | .183  (.308) | | .129  (.109) | | | |
|  | | | |  | | |  | | | | |  |  | | |  | | |  | |  | |  | |  | |  | |  | | | |
| Party Identification | | | | -.236  (.207) | | | -.235^**^  (.072) | | | | |  | -.157  (.212) | | | .065  (.074) | | |  | | -.387^+^  (.210) | | .238^+^  (.133) | |  | | -.124  (.217) | | .008  (.076) | | | |
|  | | | |  | | |  | | | | |  |  | | |  | | |  | |  | |  | |  | |  | |  | | | |
| Actual Age | | | | -.026^***^  (.004) | | | -.023^***^  (.002) | | | | |  | -.041^***^  (.004) | | | -.043^***^  (.002) | | |  | | -.042^***^  (.004) | | -.044^***^  (.003) | |  | | -.032^***^  (.004) | | -.031^***^  (.002) | | | |
|  | | | |  | | |  | | | | |  |  | | |  | | |  | |  | |  | |  | |  | |  | | | |
| Education | | | | -.553^*^  (.245) | | | -.130  (.106) | | | | |  | -.704^*^  (.255) | | | -.145  (.109) | | |  | | -.291  (.251) | | .683^**^  (.215) | |  | | .132  (.256) | | .079  (.112) | | | |
|  | | | |  | | |  | | | | |  |  | | |  | | |  | |  | |  | |  | |  | |  | | | |
| Male | | | | -.240^+^  (.138) | | | -.044  (.053) | | | | |  | -.346^*^  (.142) | | | -.011  (.055) | | |  | | -.269^+^  (.141) | | .043  (.095) | |  | | -.112  (.144) | | .137^*^  (.055) | | | |
|  | | | |  | | |  | | | | |  |  | | |  | | |  | |  | |  | |  | |  | |  | | | |
| White | | | | .760^***^  (.189) | | | .404^***^  (.079) | | | | |  | .776^***^  (.200) | | | .247^**^  (.081) | | |  | | .615^**^  (.195) | | .284^*^  (.139) | |  | | .394^+^  (.200) | | .342^***^  (.083) | | | |
|  | | | |  | | |  | | | | |  |  | | |  | | |  | |  | |  | |  | |  | |  | | | |
| Black | | | | -.577^*^  (.257) | | | -.360^***^  (.102) | | | | |  | -.295  (.273) | | | -.231^*^  (.105) | | |  | | -1.16^***^  (.277) | | -.623^**^  (.180) | |  | | -.577^*^  (.267) | | -.392^***^  (.107) | | | |
|  | | | |  | | |  | | | | |  |  | | |  | | |  | |  | |  | |  | |  | |  | | | |
| Constant | | | | 1.14^*^  (.413) | | | .841^***^  (.134) | | | | |  | 1.12^*^  (.427) | | | 1.01^***^  (.137) | | |  | | 1.41^**^  (.429) | | .596^*^  (.244) | |  | | .788^*^  (.424) | | .846^***^  (.139) | | | |
|  | | | |  | | |  | | | | |  |  | | |  | | |  | |  | |  | |  | |  | |  | | | |
| Observations | | | | 989 | | | 6213 | | | | |  | 990 | | | 6211 | | |  | | 988 | | 2007 | |  | | 988 | | 6212 | | | |
| Pseudo R^2^ | | | | .073 | | | .037 | | | | |  | .102 | | | .085 | |  | | .126 | | .089 | |  | | .080 | | .055 | |  |  |  |
|  |  |  | | |  | | |  | |  | | | |  | | | | | | | | | | | | | | | | |  |  |

Significance codes: ^+^p < .10, ^*^p < .05, ^**^p < .01, ^***^p < .001

Note: Dependent variables are recoded from 0-1, with 1 being the pro-pit position and 0 representing all other responses. CCES utilize post-stratification weights, but Lucid does not provide weights with its raw data. Sources: Pooled Lucid Surveys 2019-2021; Lucid Survey; 2018 CCES Team Module

Table A5: (OLS) Predictors of Saying it Should Be Legal to Own a Pit Bull

|  | Full Sample | Whites Only | Non-Whites | Full Sample |
| --- | --- | --- | --- | --- |
|  |  |  |  |  |
| Inner-City Treatment | -.097^***^ | -.126^***^ | -.018 | -.018 |
|  | (.017) | (.020) | (.034) | (.034) |
|  |  |  |  |  |
| White |  |  |  | .087^**^ |
|  |  |  |  | (.028) |
|  |  |  |  |  |
| White*Inner-City |  |  |  | -.108^**^ |
| Treatment |  |  |  | (.039) |
|  |  |  |  |  |
| Constant | .648^***^ | .671^***^ | .584^***^ | .584^***^ |
|  | (.017) | (.014) | (.024) | (.024) |
|  |  |  |  |  |
| Observations | 3196 | 2351 | 845 | 3196 |

Significance codes: ^+^p < .10, ^*^p < .05, ^**^p < .01, ^***^p < .001

Source: Pooled Lucid Surveys from June 2020, July 2020, August 2021

Table A6: (Logistic Regression) Predictors of State Adopting Anti-BSL Law

|  | [1] | | [2] | | [3] | | [4] | | [5] | | [6] | | |
| --- | --- | --- | --- | --- | --- | --- | --- | --- | --- | --- | --- | --- | --- |
|  |  | |  | |  | |  | |  | |  | |  |
| Racist Google Searches | | -3.96^*^ | |  | | -5.13^*^ | |  | | -6.23^*^ | |  | |
|  | | (1.84) | |  | | (2.49) | |  | | (2.78) | |  | |
|  | |  | |  | |  | |  | |  | |  | |
| White Opposition to Interracial Dating | |  | | -2.41^+^ | |  | | -2.84^*^ | |  | | -2.33 | |
| (Pew Values, 1987-2012) | |  | | (1.27) | |  | | (1.43) | |  | | (1.67) | |
|  | |  | |  | |  | |  | |  | |  | |
| White Republican Partisanship | |  | |  | | 2.48 | | 4.03^+^ | | 2.08 | | 4.57^+^ | |
| (Pew Values, 1987-2012) | |  | |  | | (2.05) | | (2.35) | | (2.15) | | (2.57) | |
|  | |  | |  | |  | |  | |  | |  | |
| White Support for Limited Government | |  | |  | | -4.32^*^ | | -3.70^+^ | | -3.95^+^ | | -4.26^+^ | |
| (Pew Values, 1987-2012) | |  | |  | | (2.04) | | (1.98) | | (2.08) | | (2.27) | |
|  | |  | |  | |  | |  | |  | |  | |
| Black Population Proportion | |  | |  | |  | |  | | 3.44 | | -2.70 | |
| (2010 Census) | |  | |  | |  | |  | | (5.17) | | (4.85) | |
|  | |  | |  | |  | |  | |  | |  | |
| Constant | | 1.23 | | .764 | | 2.83^*^ | | 1.03 | | 2.85^*^ | | 1.18 | |
|  | | (.761) | | (.620) | | (1.33) | | (.976) | | (1.31) | | (1.02) | |
|  | |  | |  | |  | |  | |  | |  | |
| Observations | | 48 | | 47 | | 48 | | 47 | | 48 | | 47 | |
|  |  | |  | |  | |  | |  | |  | |  |
| Pseudo R^2^ | .088 | | .063 | | .172 | | .135 | | .179 | | .140 | | |

Significance codes: ^+^p < .10, ^*^p < .05

Note: Dependent variable is coded 0-1, with 1 representing states who’ve adopted laws preempting local breed-specific legislation. All variable except actual Black population proportion coded from 0-1, with 1 taking on the highest state’s values and 0 representing the lowest state value. Analysis restricted to continental US due to sampling limitations in the Pew Data. Data on state laws from Fix and Mitchell (2017); state-level white opposition to interracial dating from the 1987-2012 Pew Values Survey Merged File; data on racist searches are from Google Trends, 2004-2019.
