# Supplementary material for: The racialization of pit bulls: What dogs can teach us about racial politics
Source: PLoS One. 2024 Jun 28;19(6):e0305959. doi: 10.1371/journal.pone.0305959 (PMC11213322; doi:10.1371/journal.pone.0305959)
Supplement: Attachment — Submitted filename: plos memo.docx [file pone.0305959.s003.docx]

Comments to the Editor

Thank you very much for the opportunity to revise and resubmit our manuscript with minor revisions. We have worked hard to address all the reviewers’ critiques and to incorporate their thoughtful suggestions into the revised manuscript.

More specifically, you’ll see from our memo that we have now engaged with the relevant literature that we regrettably omitted from the first submission, deemphasized the automated sentiment analyses, added some key details about measures and results in accordance with the reviewers’ advice, and further qualified our experimental findings with some new caveats. We hope that these changes have now made the manuscript suitable for publication in *PLOS ONE*.

Response to Reviewer 1

Thanks so much for the very helpful suggestions about relevant literature to incorporate into the revised manuscript. We regret these omissions from the prior submission and the revised manuscript now cites these studies in detail, paying particularly close attention to Thomspon et al (2022).

(1)R1 writes: “This is a fantastic study that has one major flaw: The authors have ignored extremely relevant literature prior research.” The reviewer goes on to note that it is “totally unacceptable” to not cite Thompson et al. (2022), as their article “developed a theory for why racial attitudes should be related to views about pit bulls and breed-specific legislation, it measured peoples’ perceptions of the racial composition of pit bull owners, it tested whether you could prime racial concerns and influence support for breed-specific legislation, and it also examined the relationship between racial attitudes and views about breed-specific legislation.”

RESPONSE: We first want to thank the reviewer for the kind words about our study. Second, we apologize for our failure to cite this important study. Not doing so was an honest omission that simply stemmed from us writing our initial draft of the manuscript long before that article was published. So, we’re very grateful that the reviewer brought these findings to our attention, and we’re pleased to highlight them prominently in our revised manuscript (more on that below).

(2) R1 relatedly writes, “True, the current submission is a MUCH better study than Thomspon et al. The former used a small sample, weaker measures, and focused only on college students. Still…the current authors need to cite Thompson et al. (2022), they need to discuss the findings in that study, and they need to explain how their study goes beyond it and offer some explanation for why the findings in it are different than the earlier ones.”

RESPONSE: We, once again, thank the reviewer for the nice words about our study and express sincere regrets over the omission of Thomspon et al. (2022) from our first submission.

We also really appreciate R1 pushing us to explain why the findings in our study may differ from theirs and now devote 1.5 paragraphs at the front-end of the manuscript to discussing these findings and offering informed explanations for why ours are different. After noting that there have been few empirical analyses testing scholarly and journalistic contentions about the racialization of pit bulls, the revised manuscript now states the following:

*“Moreover, Thompson, Pickett, and Intravia’s (2022) recent empirical study found that neither experimentally linking pit bull owners with African Americans via racial imagery, nor harboring stereotypes of pit bull owners as disproportionately Black, were associated with support for breed-specific legislation among college students.*

*Thompson et al (2022), however, conclude that study by noting the need to replicate their findings with representative samples. They specifically discuss how the young age of their college sample could affect the results if attitudes toward pit bulls have improved since Michael Vick’s 2007 dog-fighting controversy. This is a particularly prescient point. Consistent with the unusually low levels of support for breed-specific bans in Thompson et al.’s data (1.74 on a 1-5 scale), we note multiple times throughout the manuscript that age is probably the strongest predictor of public opinion about pit bulls presumably because American youths have been socialized amidst increasingly positive images of the breed on social media.* *Younger and college-educated Americans score significantly lower in racial prejudice (see discussion below), as well, which could help further explain why the link between pit bulls and African Americans did not increase support for breed-specific legislation among college students.”*

(3) R1 writes, “Given the current authors' focus on the perceived racial composition (or racial typification) of dog owners (e.g., Figure 2), I would also direct them, when talking about the racialization of crime (pp. 8-11), to the extensive work on how the racial typification of crime (i.e., the perceived racial composition of criminals) is related to support for harsh policies. The authors should start with Chiricos et al.'s (2004) seminal study and go from there in their literature review.”

RESPONSE: We regret the omission and acknowledge our embarrassing blind spot here as political scientists to some of the relevant literature in other disciplines. We are, therefore, grateful to the reviewer for alerting us to the relevant work on this topic in criminology. The Chiricos et al. piece on the racial typification of crime and support for punitive measures was particularly helpful and we now cite that study three different times in our revised manuscript. We also cite additional studies on the racialization of crime that we picked up from R1’s suggestion to look to the lit reviews in Chiricos et al. and Cullen et al. for guidance.

So, we want to conclude here by sincerely thanking the reviewer again for his help with these important additions to the revised manuscript.

Response to Reviewer 2

Thank you for both the kind words about our manuscript and for your thoughtful critiques of it. Your concerns about the automated sentiment analyses were particularly important in improving the revised manuscript.

(1) R2 writes, “I am fully persuaded by the analysis in the “Racial prejudice and public opinion” section. For one thing, I find it very hard to imagine that the relationship between racial and pit bull attitudes runs in the opposite direction. For another, I cannot think of any important omitted variable that isn’t quite closely related to something the authors already control for in some model…I think the authors could make more of the (many) reasons why we should take their correlational evidence very seriously.”

RESPONSE: We are SO very grateful to the reviewer for these comments. It is indeed difficult to imagine how these results would be susceptible to the same endogeneity issues found in the correlations between racial attitudes and support for affirmative action (Sniderman and Carmines 1997), welfare (Goren 2022), and Trump (Griffin et al. 2021). Nor is omitted variable bias a threat to the statistical significance of the findings. The relationship between racial attitudes and public opinion remained intact in the face of every model specification we tried, as there were only three consistent predictors of public opinion about pit bulls across the hundreds of regressions we’ve run--age, racial attitudes, and general favorability towards dogs--all of which are include in our multivariate analyses.

We were quite happy, then, to heed the reviewer’s advice and make more out of why we should take “the correlational evidence very seriously.” The concluding paragraph of this section in the revised manuscript now offers reasons why we’re confident that these relationships are not the product of endogeneity or omitted variable bias. The new discussion of omitted variable bias and the robustness of the findings across every model imaginable also indirectly addresses some of the concerns about model specifications raised by R3 (see below)

(2) R2 writes, “the analysis of coverage of pit bulls isn’t very convincing. It seems to me that the burden of this analysis is to show that coverage of pit bulls – and perhaps, by extension, wider cultural discourse concerning dogs – associates the breed with African Americans very prominently or regularly. But it’s only the very rare pit bull story that mentions African Americans, about 2%. I don’t know exactly how to calibrate this percentage, but it doesn’t sound very high to me. The authors need to provide some basis for thinking that this percentage indicates that associations between pit bulls and African Americans are sufficiently pervasive in the culture that we should expect views of pit bulls to be racialized.”

(3) R2 relatedly writes, “I don’t follow the authors’ analysis of dog-news sentiment. Figure 3 essentially depicts an interaction, with respect to tone, between the type of dog in a story and whether African Americans are mentioned. It seems to me that the observed interaction is the reverse of what we’d expect, under the authors’ argument. That is, if pit bulls were especially affected by association with African Americans, then I would expect the relative impact of that association on sentiment to be greater for pit bulls than for other dogs. But that’s not the case here: for non-pit bulls, association with African Americans increases the negativity of net sentiment by about 60%; for pit bulls, the comparable effect is about 40%.”

RESPONSE: These two passages above are both very fair critiques and it’s now clear to us from the reviews provided by R2 and R3 that the revised manuscript needed to overhaul how we present our automated content analyses.

To be sure, we still think the analyses in Figure 3 of the original manuscript are an important part of the story since racialized stories about pits are 20-points more negative than those that don’t explicitly reference African Americans and that there’s a larger total volume of negative racialized news stories about pit bulls than all the other dogs we looked at combined. We also think these numbers here are an enormous underestimation of negative racialized news coverage, as qualitative analyses suggest that negative associations between African Americans and pit bulls in media and pop culture occur largely through imagery--and our automated content analyses can only identify explicit references to race.

But we also completely agree with R2 and R3 that our automated content analyses just aren’t as convincing as the other quantitative analyses, and the evidence in support of H1 is not as strong as the data testing H2-H6 in the original manuscript. So, after careful consideration we decided to jettison the “Negative News Coverage” section from the revised manuscript.

Instead, the revision makes only brief reference to our “suggestive quantitative evidence” from Crimson Hexagon in the “Theoretical Background and Empirical Expectations” section. After discussing prior qualitative accounts of how media and pop-culture portraits negatively depict pit bulls when they’re linked up with African Americans in more detail than the original submission, the revised manuscript goes on to state:

*And our subsequent research provides some suggestive quantitative evidence on the heightened negativity of news coverage when pit bulls are associated with African Americans (McThomas and Tesler 2024). Drawing on automated sentiment analyses from Gary King’s analytics platform, Crimson Hexagon, we found that the net sentiment (positive minus negative) of headline news stories about pit bulls that explicitly mentioned African Americans was significantly lower (p<.001) than the already high levels of negativity in all news coverage of the breed (-68 to -48 respectively).*

We think that restructuring our discussion of media portraits this way has several benefits. First, and most importantly, deemphasizing the media findings leaves the manuscript less vulnerable to criticism since that was our weakest hypothesis test. Second, the media findings served largely to bolster empirical expectations about the link between racial attitudes and public opinion. Little is lost, then, from briefly referencing these automated content analyses in the theoretical background and empirical expectation section instead of dedicating a whole empirical section to them. Indeed, almost none of the article-length studies cited on how crime, welfare, and drug addiction have been racialized in public opinion via media portraits include original content analyses of news coverage. Third, referring the reader to the more detailed presentation of the automated content analyses from our book manuscript in progress, which includes an entire chapter on race and media portraits of pit bulls, allows us to address the critiques raised by the reviewers in much greater detail than we could do in the space allotted to a single section of an article. Finally, removing this section provides us with additional space to incorporate the reviewers’ other suggestions. The revised manuscript’s text (minus references) is nearly the exact same length as the original.

We are, therefore, most grateful to R2 and R3 for convincing us to do something we had been stubbornly reluctant to do even after receiving similar feedback from our colleagues. Upon closer reflection, we think there is a significant addition-by-subtraction effect here and that the manuscript is considerably strengthened by removing this material.

(4) R2 writes, “My other significant concern involves the experimental evidence. I accept that it supports the authors’ hypothesis (H5), but that hypothesis isn’t actually a very specific test of their argument. And other analysis of the experiment seems to threaten the authors’ interpretation of this effect in terms of racialization. Specifically, the inner-city treatment doesn’t increase the impact of racial attitudes (black favorability) on pit bull attitudes – and this is precisely the effect the authors need to establish, i.e., implicitly associating pit bulls with African Americans increases the weight of racial attitudes in pit bull attitudes.”

# RESPONSE We appreciate this valid critique, especially since it also dovetails with some of R3’s comments below. The revised manuscript, therefore, takes multiple steps to address it.

# First, we provide more reasons for why we do not think that the null interactive effect here for inner-city*Black favorability is a serious threat to interpreting the experiment in terms of racialization. In addition to our discussion of the prior studies showing that it’s a lot more difficult to experimentally prime racial attitudes in issues that are already racialized like pit bulls, we’ve incorporated R3’s comment into the revised manuscript about how interaction analyses are “extraordinarily power-hungry.” We now cite Andrew Gelman’s analysis, “You Need 16 Times the Size to Estimate an Interaction Than to Estimate a Main Effect” among others in that discussion and then use it as a segue to further highlight the significant interactive effect for inner-city*White. We think that significant interaction greatly aids the interpretation in terms of racialization, as it suggests that the inner-city treatment is tapping into something distinctly racial; we now explicitly say so in the revised manuscript.

# At the same time, though, we’ve also added some additional caveats to conclude the experimental section, which reflect the legitimate concerns raised by both R2 and R3 (see more below). We think those qualifications help insulate the analysis a bit from critiques and are therefore grateful to both reviewers for their comments on this section.

(5) R2 includes some more minor comments, as well, such as our misnumbering of hypotheses and figures; the review also notes that the wording of H4 in the original manuscript (“stereotypes of Black men as violent will be particularly potent predictors of rating pit bulls unfavorable”) is vague with regards to what is meant by “particularly potent.”

RESPOSNE: We thank the reviewer for identifying these smaller issues. The revised manuscript has fixed the misnumbering and we’ve changed the vague wording of the hypothesis to “*stereotypes of Black men as violent will be a significant predictor rating pit bulls unfavorably even after controlling for stereotypes of Black women and other groups as violent.”*

Response to Reviewer 3

Thank you very much for your careful engagement with our work and for providing us with helpful suggestions that we’ve incorporated into the revised manuscript.

(1) R3 writes, “I don’t think Figure 3 (media) supports the thrust of the argument. Stories about pitbulls mentioning African Americans are more negative – but so are stories about non-pitbulls. This could be straightforward anti-Black racism in the media, with no special link to pitbulls. And pitbull stories are more negative than other dogs, with or without mentioning African-Americans, consistent with pitbulls being (perceived to be) more violent regardless of their owners. Looking at the appendix, stories with Rottweilers are more likely to mention Black people then those with pit bulls! This doesn’t mean the theory is necessarily false given the potential implicit associations etc, but it does need to be acknowledged that this evidence is perhaps suggestive, but not watertight.”

RESPONSE: As mentioned above, we are especially grateful to both R2 and R3 for raising these issues about our automated content analyses. Those comments resulted in the biggest change to our revised manuscript—jettisoning the section on “Negative News Coverage,” and instead briefly mentioning our “suggestive quantitative evidence” from Crimson Hexagon in the “Theoretical Background and Empirical Expectations” section. We discussed above the multiple ways that doing so improves the manuscript and want to thank R3 for helping us do so. We also particularly appreciated the reviewer’s suggestion to present this evidence as suggestive instead of watertight. Not only does the revised manuscript do this in its brief presentation of the findings but we now also take that recommended approach in our book manuscript’s chapter on race, media, and pit bulls.

(2) R3 provides a number of helpful suggestions for how to think about and address the null interaction between Black favorability*inner-city in the experimental section. The review first notes the possibility of ceiling effects for experiments trying to prime something that is already salient. But R3 also raises the possibility that “Perhaps this is because the results are actually not about race. Maybe people think its ok to own large and aggressive dogs out in rural issues, where there is lots of space…Or perhaps this about location…Or measurement issues, due to blunt racial attitudes…Maybe its just power: interaction analyses are extraordinarily power-hungry.”

# RESPONSE: We really appreciate these comments, especially since they echo similar thoughts from R2. The revised manuscript, therefore, tweaks the experimental section in the following ways.

# First, we include some qualitative research in this section for context about how/why the inner-city frame closely maps onto how the pit bull is thought to have been racialized, citing Dickey’s (2017) contention that “Once the pit bull was portrayed as an ‘inner-city dog’…it became a magnet for racial fears about crime and the American underclass.” Second, we provide more reasons for why we do not think that the null interactive effect here for inner-city*Black favorability is a serious threat to interpreting the experiment in terms of racialization. In addition to R3’s comments on the difficulty of experimentally priming racial attitudes in issues that are already racialized like pit bulls, we’ve incorporated the reviewer’s comment into the revised manuscript about how interaction analyses are “extraordinarily power-hungry.” We now cite Andrew Gelman’s piece, “You Need 16 Times the Sample Size to Estimate an Interaction Than to Estimate a Main Effect” among others in that discussion and then use it as a segue to further highlight the significant interactive effect for inner-city*White. We think that significant interaction greatly aids the interpretation in terms of racialization, as it suggests that the inner-city treatment is tapping into something distinctly racial; we now explicitly say so in the revised manuscript.

# But we are also responsive to R3’s concern that maybe the results aren’t actually about race here. So, after making the case for why we still think they are, we conclude this section with the following two-sentence paragraph:

*“**Yet while the results are consistent with both that interpretation and prior research on how racialization erodes white support for issues and policies associated with African Americans, it is important to conclude here by noting that we cannot say for certain what the exact mechanism is behind why pit bulls are less popular when they’re framed as inner-city dogs. It’s possible, for instance, that there’s less support for large, muscular, dogs in cities because people think they need more space to flourish. So, we hope that future research will build on these results by further examining how and why implicit associations with African Americans erode whites’ support for legalizing pit bulls.”*

# (3) R3 asks, “Why are only whites included in the surveys starting on p.15? Surely the attitude structure of non-whites is the same: they associate African-Americans with pitbulls, so their views toward them is partly a function of views toward African-Americans. If its specifically anti-black views that matter here, there are certainly Latino and Asian Americans with anti-black views.” The reviewer relatedly goes on to state, “I think the authors should include all respondents, including non-white ones. Or if they have some strong theoretical reason for excluding them, at least a set of models including them in in the appendix and include a footnote telling the reader the results.”

RESPONSE: This is a fair critique, and we should have justified restricting the racial attitude analyses to just whites in the original manuscript. So, the revised manuscript now notes, “*Like most of the research we cited in the previous section on how racial attitudes are associated with public opinion about such issues as welfare, crime, drug addiction, health care, government spending and affirmative action, our analyses in this section are limited to whites.*” We prefer to keep the analysis limited to whites in large part for consistency with the prior literature we’re engaged with, and quite frankly because Tesler received some friendly fire from a few of these scholars for pooling races together for the racial attitude analyses in *Obama’s Race*.

But we’re also very happy to take R3’s advice of including the pooled models in Table A4 of the appendix and referring the reader to them when noting that neither the statistical nor substantive significance of the findings is affected by restricting the sample to just whites.

(4) R3 writes, “Justify the control variables. There is an awful lot of research arguing partisanship is a product of racial attitudes. I’m not sure what including attitudes toward whites would do to the model, but its not clear what its purpose is. And there is no control for income, which is pretty standard and also correlated with dog ownership…I think the authors should do a bivariate model, and report if the results are very different or not, and if so then which control variables make the big difference.

RESPONSE: This, too, is a very fair point, and the original manuscript should’ve included a greater discussion of why we included the control variables we used in the model. The revised manuscript now provides such a discussion in the following paragraph:

“*Consistent with H1’s expectations, unfavorable views of African Americans and OFR were significantly correlated with our four questions about pit bulls in both the CCES and Lucid data (see Table A1 of the appendix). But it’s important to account for other variables to test H1’s contention that racial prejudice is an independent predictor of public opinion about pit bulls. Our findings are robust to every possible specification we tried, so we selected our final model based on several factors. We followed Winter (2008) and included favorability ratings of whites alongside favorability of African Americans to assess the relative influence of each variable while holding the other one constant. Partisanship is in the model to both account for its growing association with racial attitudes (e.g. Tesler 2016; Griffin et al. 2021) and to determine the degree to which public opinion about pit bulls is rooted in party polarization. Our earlier discussion of pit bulls and masculinity prompted us to control for sex. We included education to control for its well-documented association with racial attitudes (correlation with OFR =* ***-****.17), and to assess how socioeconomic status is linked to public opinion about the breed. Finally, and most importantly, we included age to account for the fact that young people score significantly lower in racial prejudice (age is correlated with ORF at r = .28) and have much more favorable opinions of pit bulls than older Americans*.”

You can also see that the start of this paragraph heeds R3’s advice and refers the reader to the table of bivariate coefficients in the appendix. As we now note in the revised manuscript, all our dependent variables are significantly linked to both Black favorability ratings and old-fashioned racism in the bivariate models. The concluding remarks for this section further note, in accordance with R2’s suggestion, how robust the models are to alternative model specifications (e.g. including income, homeownership, region, etc); and reiterates that the most important control variable is age since that is significantly linked to both racial attitudes and public opinion about pit bulls.

Smaller issues raised by R3

(5) R3’s smaller issues section raises a couple of valid questions about measurement. First, R3 writes, “Research on racialization, such as Tesler and others, has emphasized that those with positive (pro-Black) racial attitudes become more positive toward racialized issues. Is that the case here? Similarly, the paper makes various claims, such as “the racialization of pit bulls has eroded public support for the breed”. Has it? Or has it just made some people like them more, and others less.”

RESPONSE: This is an excellent question that the revised manuscript addresses in a bit more detail than the original. One of the reasons we went with more blatant racial attitude measures (favorability, old fashioned racism, and stereotypes) is that we were primarily interested in how the racialization of pit bulls has eroded support for the breed. Our prior research shows that those blatant measures are unable to identify racially sympathetic whites since the low-end lumps together actively pro-Black racial liberals with colorblind whites (Tesler and Sears 2015; Tesler 2016). We now make that point clearer when discussing our measures.

Consistent with that explanation, the predicted results from Table 1 show that whites with the most favorable ratings of African Americans still do not rate pit bulls higher than other dog breeds, while those with the least favorable views of African Americans rate pit bulls *much* lower than other dog breeds (-5 vs. +53 respectively). The revised manuscript now makes note of that point to help further justify the claim that the racialization of pit bulls has eroded public support for the breed.

(6) R3 relatedly writes about measurement, “Its good to report on the discussion of the racial attitude measure, that its less correlated with other social attitudes etc. Its not obvious that this is a good thing, or that it will result in conservative estimates. What we really want is a true measure of racial attitudes. I’m no expert, but I wouldn’t assume attenuation bias, since the error isn’t random just noise, its concentrated at one end of the scale. The authors could look carefully at the methodological research on this. Or, they could just point out the properties of the measures used, and say they aren’t sure exactly what effects this could have.”

RESPONSE: This, too, is a fair point. We were working off the assumption here that social desirability pressures to rate groups equally results in misclassifying racially prejudiced whites; and if so, that would likely attenuate the relationship. However, since the reviewer isn’t so sure and since we have not engaged in the same methodological inquiry as we have in the past with the low-end of these scales, we’ve proceeded with caution and removed the discussion of why these are probably conservative estimates from the revised manuscript.

(7) R3 asks, “What are the potential generalizability of these findings? As I’m writing this, I noticed that the UK is going to ban the related breed “American XL Bully”. And after some quick googling, it seems like in other countries including Australia and Canada there are also debate about bans on pitbulls. The authors may not be claiming direct evidence, but given that anti-Black racism and pitbull bans both occur in many countries, it seems worth discussing.”

RESPONSE: This is another terrific question that we hope to actively address in our book with data from YouGov-UK. We suspect the relationship will hold for the reasons R3 mentions. But we’d prefer to punt on the portability to other countries in the current manuscript because we just don’t have the evidence to support that claim right now.

(8) R3 writes, “My own experience with lucid is the data is quite low quality, worse than other online platforms. What kind of measure did the authors take? Include that somewhere (unless I missed it). Does excluding low quality respondents change the results?”

RESPONSE: We regrettably did not include any attention checks in our Lucid surveys. However, we are reassured by the fact that both the intercepts and the relationships between all our DVs and racial attitudes look similar in the Lucid and the YouGov/CCES data. The CCES relationships tended to be a bit stronger, too, which makes us think that if inattentive Lucid respondents are affecting our results, they’re likely introducing a conservative bias into our estimates. To help reassure the reader, the revised manuscript now notes that we replicate our findings with the higher quality YouGov/CCES sample to make sure that our results aren’t merely an artifact of poor data quality in the Lucid surveys.
