# Supplementary material for: The racialization of pit bulls: What dogs can teach us about racial politics
Source: PLoS One. 2024 Jun 28;19(6):e0305959. doi: 10.1371/journal.pone.0305959 (PMC11213322; doi:10.1371/journal.pone.0305959)
Supplement: Attachment — Submitted filename: plos memo2.docx [file pone.0305959.s004.docx]

Response to the Editor

Thank you for the opportunity to revise and resubmit the manuscript. We are especially appreciative that you plan to make a quick decision upon receipt of the revised manuscript without another round of reviews.

We were also particularly pleased to see that R1 and R2 described our response to their critiques as “phenomenal” and “great” respectively, and that R6 described the topic as “wonderful, creative, and necessary.”

As the memo details, we have restructured the revised manuscript in accordance with your suggestion to have a new section that provides greater detail up front on the data and methods used to test our hypotheses. While we were initially reluctant to make this change, we think it has improved the manuscript and hope that these revisions, combined with the other reviewers’ evaluations, have now made the article suitable for publication in PLOS ONE.

Response to Reviewer 4

We thank the reviewer for pushing us to provide a more detailed standalone section on the data and methods used in our manuscript. Doing so helped strengthen the revised article.

(1) R4 first writes, “in the abstract they list 5 points which they believe they demonstrate. However, not until later in the manuscript do they start addressing what appears to be the five hypothesis, H1 to H5, which this reader assumes are the major goals of the paper. They should have been formerly stated as hypotheses or at least the opposite or alternatives of five null hypotheses at the start of a formal statistical analysis plan.”

While our five theoretically informed hypotheses were all formally stated in the “Theoretical Background and Empirical Expectations” section of the prior manuscript, it is certainly reasonable for the reviewer to ask for a more detailed standalone section on the data and methods used to test those expectations.

The revised manuscript, therefore, now includes a new “Data and Methods” section following the section on “Theoretical Background and Empirical Expectations.” This new section details the various data, measures, and statistical analyses used to test our formal hypotheses. We think that these revisions have improved the manuscript and thank both the reviewer and the editor for pushing us to make them.

(2) In addition, R4 writes, “there appears to be some relevant information [missing]. For example Figure 1 is presented with percentages which is fine. However, as mentioned above, the authors give their take on the results descriptively. In the last bar presentation ‘Rate pit bulls very favorably’ how significant is that two percentage point difference. Probably statistically so with the sample size, but how meaningful practically is that difference?”

We’d like to respectfully note here that the statistical and substantive significance of this difference were addressed on pg 6-7 of the prior manuscript where we write: "There were not significant differences between the races in how favorably they rated pit bulls; but after controlling for the fact that white people have more favorable views about dogs than Black people do, and that attitudes about dogs in general strongly predict attitudes towards pit bulls (see Table 1), African Americans were significantly more likely than whites to rate pit bulls very favorably (40% to 31% respectively)."

(3) On page 8 below Figure 2, R4 writes, “the authors note that, ‘It’s hardly a coincidence, either, that the two dog breeds stereotyped as Black-owned are the two breeds that evoke the most fear by far from the public (Tesler 2020).” Has fear and type of breed been statistically associated in the reference?’

The reviewer asks a very fair question here. So, the revised manuscript now elaborates on this point by adding the following information on pg. 8 about the link between fear and breed provided in this reference:

“Over 40 percent of respondents in a 2018 Lucid survey we conducted said that “scary” described pit bulls and rottweilers ‘extremely’ or ‘very well’ (46 and 41 percent, respectively). But only about 10 percent said the same thing about golden retrievers, collies, Dalmatians and Labradors. These four breeds were all rated at least 15 percentage points more favorably than rottweilers and pit bulls in our surveys as well (Tesler 2020).”

(4) R4 further writes, "Also throughout the manuscript the words, ‘suggest’ and ‘implicit’ appear, which gives the impression of possible author bias, which I’m sure is not the case. See page 22, “The results in Figure 4 show that implicitly associating African Americans with pit bulls via the “inner city” treatment also significantly decreased white Americans’ support for legalizing the breed.”

We were a bit perplexed by this comment, as our repeated use of “implicit” simply refers to the long line of social science literature cited in the article on the impact of messages that indirectly associate a racial group with a policy/person/dog with racially evocative words (e.g. inner-city) without making an explicit reference to that group by name.

To avoid any potential confusions about the meaning of “implicit” here, we now make further note of this distinction between implicit vs. explicit racial references on pg 13, writing, “Several experiments, which randomly assigned subjects to receive *implicit* racialized messages (e.g., racial images and/or race-coded language that doesn’t *explicitly* reference a particular racial group) about specific issues, have affected white Americans’ opinion about crime, welfare, drug treatment, gun control, government spending, education programs, Social Security, the minimum wage, the Iraq War, and the coronavirus pandemic.”

(5) R4 writes, “On Table A3 “Old fashion Racism” is a variable. How strongly does that really associate with choosing a dog? There are no measures of concordance given with the logistic presentations. So the strength of the association is not clear. Like wise [sic] on Table 1, where are the R-square or partial r-square measures?

These are fair questions that we thank the reviewer for raising. The revised manuscript therefore includes R-squared or Pseudo R-squared measures in our regression tables.
